# Antiviral and Inflammatory Cellular Signaling Associated with Enterovirus 71 Infection

**DOI:** 10.3390/v10040155

**Published:** 2018-03-28

**Authors:** Yuefei Jin, Rongguang Zhang, Weidong Wu, Guangcai Duan

**Affiliations:** 1 Department of Epidemiology, College of Public Health, Zhengzhou University, Zhengzhou 450001, China; jinyuefeixuanchuan@163.com (Y.J.); zrg@zzu.edu.cn (R.Z.); 2 Department of Occupational and Environmental Health, School of Public Health, Xinxiang Medical University, Xinxiang 453003, China; wdwu2013@126.com

**Keywords:** EV71, antiviral innate immunity, cellular signaling, immune evasion, inflammatory response

## Abstract

Enterovirus 71 (EV71) infection has become a major threat to global public health, especially in infants and young children. Epidemiological studies have indicated that EV71 infection is responsible for severe and even fatal cases of hand, foot, and mouth disease (HFMD). Accumulated evidence indicates that EV71 infection triggers a plethora of interactive signaling pathways, resulting in host immune evasion and inflammatory response. This review mainly covers the effects of EV71 infection on major antiviral and inflammatory cellular signal pathways. EV71 can activate cellular signaling networks including multiple cell surface and intracellular receptors, intracellular kinases, calcium flux, and transcription factors that regulate antiviral innate immunity and inflammatory response. Cellular signaling plays a critical role in the regulation of host innate immune and inflammatory pathogenesis. Elucidation of antiviral and inflammatory cellular signaling pathways initiated by EV71 will not only help uncover the potential mechanisms of EV71 infection-induced pathogenesis, but will also provide clues for the design of therapeutic strategies against EV71 infection.

## 1. Introduction

**Figure 1:**
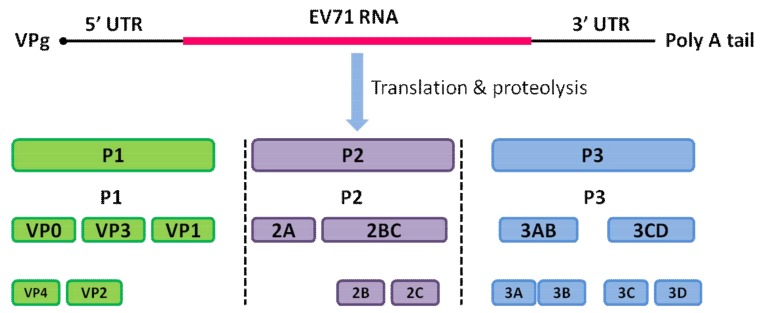
Structure of the enterovirus 71 (EV71) genome. The highly structured 5′ UTR and 3′ UTR followed by the poly (A) tail control the viral protein translation by binding to the viral VPg protein. This is followed by proteolytic cleavage, and the original protein is matured into four structural proteins, VP1–VP4, and seven non-structural proteins, 2A–2C and 3A–3D.

Enterovirus 71 (EV71), a member of the Picornaviridae family, is known to cause hand, foot, and mouth disease (HFMD) worldwide [1,2]. The viral particle of EV71 consists of a non-enveloped capsid surrounding a core of single-stranded, positive-polarity RNA of approximately 7.5 kb in size, which encodes the structural protein P1 and the non-structural proteins P2 and P3 [1,3]. The cleavage of P1 by the viral protease 3CD leads to the release of the capsid proteins VP0 (VP4 + VP2), VP3, and VP1, which subsequently leads to the assembly of the viral capsid. The cleavage of P2 and P3 by the viral protease leads to the release of seven non-structural proteins: 2A–2C and 3A–3D. The structure of the EV71 genome is shown in Figure 1. The viral capsid is composed of 60 identical protomers, each consisting of the four structural proteins: VP1–VP4 [1,3]. The proteins VP1, VP2, and VP3 of EV71 are largely exposed on the outer surface of the capsid, while the protein VP4 together with the N-terminus of VP1 decorate the inner surface of the capsid shell in a mature virion [3,4].

When an EV71 infection occurs, inflammation is known to play a critical role in the development of the host infection. This is always characterized by an infiltration of inflammatory cells, a release of pro-inflammatory cytokines and chemokines, edema, and vascular leakage [5]. It has been widely appreciated that EV71 infection can result in complex inflammation on the sites of EV71 replication accompanied by immune evasion, multiple immune cell responses, and proinflammatory cytokine release [1,6,7,8,9,10]. There have also been successes in antiviral and anti-inflammatory therapies in vivo for clinical patients with an EV71 infection [11,12]. It has been reported that many cellular signaling pathways are involved in EV71 replication and EV71-induced inflammatory pathogenesis [6,11,13,14]. Hence, characterization of EV71-induced cellular signaling is expected to provide useful information for uncovering the mechanisms of the complicated outcomes of HFMD, and aid in the design of therapeutic strategies against EV71 infection. In this review, we mainly focus on the major cellular signaling pathways involved in EV71-induced antiviral innate immune and inflammatory responses.

## 2. Oxidative Stress Mediates EV71-Induced Signaling

**Figure 2:**
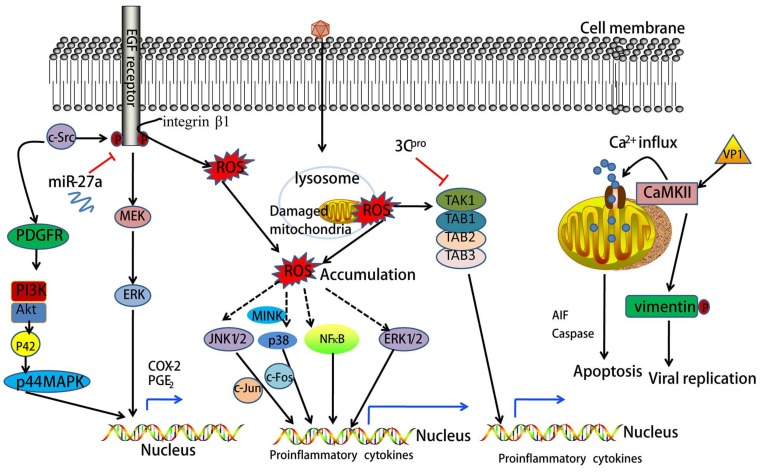
EV71 induces multiple cellular inflammatory signals and Ca^2+^-dependent signals. Epidermal growth factor receptor (EGFR)-dependent signaling plays an important role in the pathogenesis of inflammation through regulation of pro-inflammatory genes. EV71 infection induces cyclooxygenase-2 (COX-2) and prostaglandin E2 (PGE_2_) overexpression through the c-Src/EGFR/MEK/ERK and c-Src/PDGFR/PI3K/Akt/p42/p44MAPK signal pathways. The activation of EGFR can be blocked by miR-27a. EV71 infection causes reactive oxygen species (ROS) accumulation through the integrinβ1/EGFR signals and mitochondrial damage. ROS activate JNK1/2, p38, ERK1/2, and nuclear factor-kappa B (NF-κB) directly or indirectly, and further regulate the production of multiple proinflammatory cytokines. EV71-encoded 3C protease (3C^pro^) inhibits pro-inflammatory cytokine gene transcription through interactions with TAK1/TAB1/TAB2/TAB2 complexes. EV71 initiates Ca^2+^-dependent signaling, and Ca^2+^ influx. Additionally, EV71 induces host cell apoptosis by eliciting the apoptosis-inducing factor (AIF), Caspase-independent apoptotic pathway in mitochondria. The EV71 protein VP1 activates calmodulin-dependent protein kinase II (CaMKII) and further phosphorylates Vimentin, which facilitates EV71 replication.

Oxidative stress (OS) is known to take place when the generation of reactive oxygen species (ROS) overwhelms cellular antioxidant defenses [15]. Accumulated evidence indicates that ROS play a central role in the determination of the fate of a cell as second messengers which modify various signaling molecules [16]. EV71-induced generation of ROS is essential for viral replication in host cells [7,17]. A previous study has demonstrated that mitochondria are a major source of ROS in EV71-infected cells [17]. Glucose-6-phosphate dehydrogenase (G6PD) deficient cells, which have limited antioxidant capacity, exhibit susceptibility to EV71 replication and have a greater cytopathic effect (CPE), loss of viability, and upregulation of nuclear factor-kappa B (NF-κB) signaling [18]. Some in vitro intervention experiments confirm that EV71 infection-induced ROS production can activate multiple inflammatory signaling pathways. *Schizonepeta tenuifolia* Briq, a herbal constituent of traditional Chinese medicine, was shown to inhibit ROS formation and the resultant p38 kinase activation induced by EV71 infection [19]. Curcumin was also reported to reduce the production of ROS induced by EV71 infection and ROS-mediated activation of extracellular regulated protein kinases (ERK) [20]. Additionally, it was demonstrated that apigenin treatment suppresses intracellular ROS production and Jun N-terminal kinase (JNK) activation induced by EV71 infection [21]. Based on these studies, ROS formation is likely to be one biological mechanism leading to EV71 infection-induced inflammatory response (see Figure 2). In terms of these results, antioxidant drugs may be useful to treat EV71 infection-associated HFMD. Of course, these studies are also controversial.

## 3. EV71-Encoded Proteases Block Retinoic Acid-Inducible Gene I (RIG-I)-Like Receptor (RLR)-Dependent Antiviral Signaling

**Figure 3:**
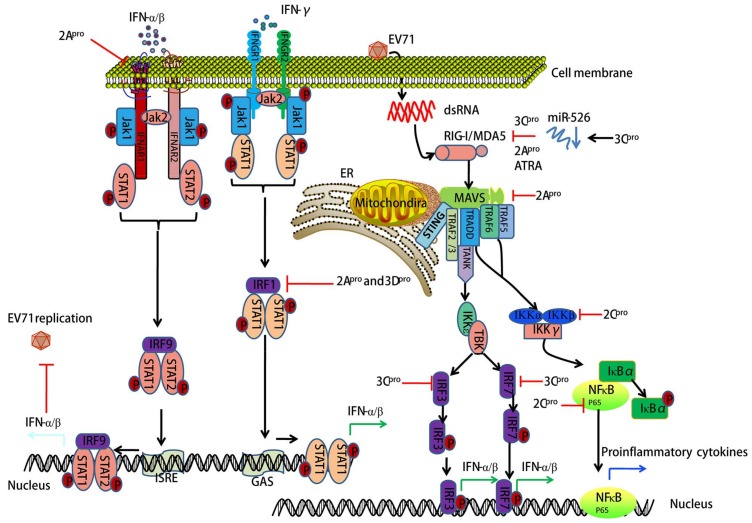
EV71 inhibits host antiviral innate immunity by targeting IFN, interferon regulatory factor (IRF), and retinoic acid-inducible gene I (RIG-I)-like receptors (RLR)-mediated signaling pathways. Antiviral innate immunity plays a critical role in EV71 infection-induced pathogenesis. EV71-encoded 2A^pro^, 3D^pro^, 3C^pro^, and 2C^pro^ inhibit IFN/STAT (signal transducer and activator of transcription)-mediated type I IFN responses by blocking IFNAR1, IRF-dependent signaling, and by targeting RIG-I, MDA5, MAVS (mitochondrial antiviral-signaling adaptor protein), IKKβ, and NF-κB (p65). On mitochondrial membranes, MAVS is regulated by RLRs such as RIG-I and MDA5, which then interact with the stimulator of interferon genes (STING), the TNF receptor associated factor (TRAF) 2/3, the TNF receptor type 1-associated DEATH domain protein (TRADD), TRAF6, and TRAF5. This process further regulates downstream IκB kinase (IKK)ε/TBK1 and canonical NF-κB signaling. Canonical NF-κB signaling occurs as the IKK complex, consisting of IKKα, IKKβ and IκBα, resulting in the proteasomal degradation of NF-κB inhibitor-α (IκBα) and thus liberating NF-κB to translocate into the nucleus and initiate pro-inflammatory cytokine gene expression. 3C^pro^ suppresses RIG-I-dependent innate immune responses through down-regulating miR-526. All-trans retinoic acid (ATRA) is a retinoic acid receptor-a (RAR-a) antagonist, which promotes RIG-I signaling against EV71 infection. ER, endoplasmic reticulum.

RLRs are cytoplasmic sensors for recognizing double-stranded RNA (dsRNA) during antiviral innate immunity in mammalian cells. Thus far, three members of RLRs have been identified: RIG-I, MDA5, and LGP2 (laboratory of genetics and physiology 2), which all have similar structures. MDA5 and RIG-I consist of three distinct domains: an N-terminal tandem caspase activation and recruitment domain (CARD), a central DExD/H box RNA helicase domain, and a C-terminal domain (CTD) [22,23]. The CARDs are used to interact with the mitochondrial antiviral-signaling adaptor protein (MAVS) to trigger downstream signaling. LGP2 lacks this domain and fails in signal transduction. The main function of the latter two domains is to recognize viral dsRNA and initiate antiviral responses through the transcription of interferon (IFN)-α/β [24]. Emerging evidence suggests that innate immune responses elicited by an EV71 infection are modulated by RLR-dependent mechanisms [25,26,27,28]. EV71-encoded 3C protease (3C^pro^) has been shown to inhibit RIG-I-mediated IFN-α/β response, which may contribute to the pathogenesis of EV71 infection [25]. Another study indicates that EV71 employs EV71-encoded 2A^pro^ to proteolytically target MDA5, further blocking IFN-α/β transcription [29]. It is known that microRNAs play a key role in the regulation of innate immune response in multiple cell types [30]. Xu et al. found that EV71-encoded 3C^pro^ inhibited RIG-I-dependent innate immune response through the down-regulation of miR-526a [27]. All-trans retinoic acid (ATRA), a retinoic acid receptor-a (RAR-a) antagonist, was reported to act on RIG-I signaling against EV71 infection [31]. Whether ATRA is beneficial for EV71 infection-associated HFMD should be demonstrated in the near future. One member of the arrestin family, ARRDC4, has a critical role in glucose metabolism and G-protein-coupled receptor (GPCRs)-related physiological and pathological processes [32]. Meng et al. reported that ARRDC4 interacts with MDA5 via the arrestin-like N domain, and further recruits TRIM65 to enhance the K63 ubiquitination of MDA5, resulting in the induction of proinflammatory cytokines during EV71 infection [33]. The interaction between ARRDC4 and MDA5 may provide a novel strategy in antiviral drugs development. Taken together, RIG-I signaling blocked by EV71-encoded 3C^pro^ and 2A^pro^ provides the molecular mechanism for innate immune evasion during EV71 infection (see Figure 3).

## 4. EV71-Encoded Proteases Inhibit MAVS-Mediated Antiviral Signaling

MAVS is comprised of 540 amino acids that form three functional domains: an N-terminal CARD (amino acids 10–77), a proline-rich region (amino acids 107–173), and a C-terminal transmembrane segment (amino acids 514–535) [34]. The CARD of MAVS interacts with the CARDs of RIG-I and MDA5, and this interaction is essential for the activation of nuclear factor-kappa B (NF-κB) and the interferon regulatory factor (IRF) signaling pathways for the regulation of pro-inflammatory cytokine and IFN-α/β production. Activation of IFN signaling has potent antiviral and growth-inhibitory effects. Previous studies have presented convincing evidence that EV71-derived protease interacts with MAVS-dependent downstream signals. For example, EV71-derived protease 2A^pro^ was shown to suppress IRF3 signaling by cleaving MAVS, which is followed by the shutdown of IFN-α/β production [29]. Similarly, another study showed that during EV71 infection MAVS was cleaved by EV71 2A^pro^ and released from the mitochondria, leading to the inhibition of antiviral IFN response [35]. Thus, MAVS-mediated antiviral signaling provides the molecular mechanisms for innate immune evasion during EV71 infection (see Figure 3).

## 5. EV71 Infection-Associated IFN Signaling

Type I IFNs (IFN-α/β) and type II IFN (IFN-γ) are widely expressed cytokines. These cytokines form the first line of defense against viral infections and also have important roles in immunosurveillance for malignant cells [36]. IFN-α/β bind to common cell-surface receptors, known as the type I IFN receptors (IFNAR1 and IFNAR2), while IFN-γ binds to different type II IFN receptors (IFNGR1 and IFNGR2) [36]. The binding of IFN-α/β to the type I IFN receptors or IFN-γ to the type II IFN receptors results in the rapid autophosphorylation and activation of Janus-activated kinase (Jak)/signal transducer and activator of transcription (STAT) pathways [37]. Activation of STATs is a common response to IFN-α/β binding [37]. Interestingly, it has been reported that EV71 infection could block IFN-mediated phosphorylation of STAT1, STAT2, Jak1, and Tyk2 through EV71-2A^pro^-induced IFNAR1 reduction [38]. Another study showed that EV71-encoded 2A^pro^ and 3D^pro^ block IFN-γ-induced IRF1 transactivation following a loss of STAT1 nuclear translocation. However, no influence was detected in IFN-receptor expression [39]. Type I and II IFN receptor deficiency increases the mortality of mice upon EV71 infection [40]. Several targets of EV71 infection such as RIG-I, MDA5, MAVS, toll-like receptor (TLR)9, TLR7 and miR-146a were also reported to interact with Type I IFN responses [13,25,29,41]. Thus, documented evidence suggests that IFN-mediated signaling pathways play an important role in host antiviral innate immunity against EV71 (see Figure 3).

## 6. EV71 Interacts with IRF Signaling

The IRF family consists of nine members in mammals, which were identified in the late 1980s in the context of research into the type I IFN system [42]. Numerous studies over the past two decades have shown the versatile and critical functions of IRFs [42]. Indeed, IRF signaling-mediated type I IFN and IFN-inducible genes play central roles in the response to pathogen-derived danger signals [43]. The 3C^pro^ of EV71 has been demonstrated to block RIG-I-mediated IRF3 activation and IFN-α/β production [25]. Similarly, EV71 inactivates IRF3 and drastically suppresses IFN-stimulated gene expression [26]. On the contrary, one study showed that IRF3 was activated and translocated into the nucleus in HT-29 cells after EV71 infection [44]. Additionally, EV71-encoded 3C^pro^ has been proposed to mediate cleavage of IRF7 rather than IRF3 [45]. These inconsistencies remain to be clarified. Taken together, IRF signaling is involved in EV71 infection-induced host antiviral innate immunity (see Figure 3).

## 7. EV71 Triggers NF-κB Signaling

NF-κB is a transcription factor of the Rel family that consists of five members in mammalian cells, e.g., RelA (p65), RelB, Rel (c-Rel), NF-κB1 (p50/p105), and NF-κB2 (p52/p100) [46]. Generally, in response to extracellular or intracellular sensors, NF-κB family proteins integrate multiple stress stimuli to modulate gene expression events that further act on cell survival, differentiation, proliferation, adhesion, immunity, and inflammation [47,48]. Emerging evidence has indicated that NF-κB signaling is essential for EV71 replication and EV71-induced inflammatory responses [49,50,51,52]. EV71 infection activates NF-κB signaling and further regulates multiple inflammatory cytokines in different cell types [49,50,51,52,53]. In vascular smooth muscle cells, EV71-stimulated degradation of NF-κB inhibitor-α (IκBα) and translocation of NF-κB into the nucleus and resultant vascular cell adhesion molecule-1 (VCAM-1) mRNA expression was blocked by a selective NF-κB inhibitor (helenalin) [52]. EV71 2C^pro^ inhibits NF-κB activation by either binding to the IPT domain of p65 and reducing the formation of heterodimer p65/p50 [54] or through association with IKKβ [54]. Additionally, resveratrol—a major active component in Polygonum cuspidatum with anti-inflammatory, antioxidant, and antitumor functions—was reported to inhibit EV71 replication and cytokine secretion (e.g., IL-6 and TNF-α) in rhabdomyosarcoma (RD) cells through blocking IKKs/NF-κB activation [55]. Thus, NF-κB signaling plays a critical role in EV71-induced inflammatory response and provides a potential antiviral strategy (see Figure 2 and Figure 3).

## 8. EV71 Interacts with TLR Signaling

**Figure 4:**
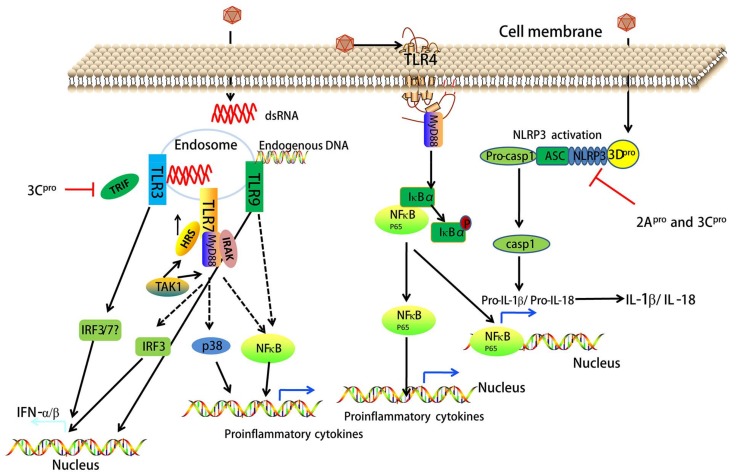
EV71 directly influences antiviral innate immunity and inflammatory responses by targeting pattern recognition receptors (PRRs). TLRs and NOD-like receptors (NLRs) belong to PRRs that sense lipids, lipoproteins, or peptidoglycans from bacteria, fungi, or protozoa, namely pathogen-associated molecular patterns (PAMPs). Additionally, these PRRs sense bacterial and viral nucleic acids, termed damage-associated molecular patterns (DAMPs). Endosomal TLR3 and TLR7 sense dsRNA of EV71. 3C^pro^ inhibits TLR3-mediated type I IFN production by blocking Toll/IL-1 Receptor domain-containing adaptor inducing IFN-β (TRIF). The interaction between hepatocyte growth factor-regulated tyrosine kinase substrate (HRS) and TAK1 regulates the ESCRT-0 complex and endosomal sorting of membrane proteins. HRS facilitates TLR7/NF-κB/p38 and TLR7/NF-κB/IRF3 signaling to produce proinflammatory cytokines and interferons during EV71 infection. Unlike TLR3 and TLR7, TLR9 senses endogenous DNA, and then mediates type I IFN production and NF-κB activation. EV71 infection also activates TLR4/myeloid differentiation primary response 88 (MyD88)-dependent NF-κB signaling, which induces multiple proinflammatory cytokines including pro-IL-1β and pro-IL-18. 3D^pro^ controls the assembly of the NLRP3 inflammasome that consists of pro-caspase-1 (casp1), ASC, and NLR family pyrin domain containing protein-3 (NLRP3). When activated, pro-casp1 matures into casp1 that cleaves pro-IL-1β and pro-IL-18 to become the mature IL-1β and IL-18 proteins. 2A^pro^ and 3C^pro^ can inhibit NLRP3 inflammasome activation.

Recent studies have provided strong evidence that the EV71-induced innate immune response is mediated by the engagement of specific TLRs [13,44]. Currently, 13 mammalian TLRs, expressed on the cell surface or in the endoplasmic reticulum (ER)/endosomal compartments have been identified. Cell surface TLRs (e.g., TLR1, TLR2, TLR4, TLR5, TLR6 and TLR11) sense lipids, lipoproteins, or peptidoglycans from bacteria, fungi, or protozoa, namely pathogen-associated molecular patterns (PAMPs). Intracellular TLRs (e.g., TLR3, TLR7, TLR8 and TLR9) detect bacterial and viral nucleic acids, termed damage-associated molecular patterns (DAMPs) [56]. An in vitro study showed that TLR/Toll/IL-1 Receptor domain-containing adaptor inducing IFN-β (TRIF)-signaling is essential to induce IFN-α/β in human intestinal epithelial cells infected with EV71 [44]. EV71 3C^pro^ inhibits the induction of innate immunity by blocking TRIF in response to TLR3 activation [26]. EV71 virus-like particles trigger the activation and maturation of dendritic cells (DCs) by inducing IκBα degradation and NF-κB activation through TLR4 [57]. Song et al. reported that increased EV71 replication induced by autophagy in 16HBE cells promotes the degradation of the endosome and leads to the suppression of the TLR7-mediated type I IFN response [41]. Hepatocyte growth factor-regulated tyrosine kinase substrate (HRS) regulates the ESCRT-0 complex and endosomal sorting of membrane proteins. HRS facilitates the TLR7/NF-κB/p38 and TLR7/NF-κB/IRF3 signaling pathways to produce proinflammatory cytokines and interferons during EV71 infection, which leads to the induction of inflammatory immune responses [58]. In vitro and in vivo studies have demonstrated that TLR9 signaling can provide protection against EV71 infection in response to DAMPs (endogenous DNA) [13]. Moreover, a related study found that the expression levels of TLR3, TLR4, TLR7, and TLR8 mRNA in peripheral blood mononuclear cells (PBMCs) of children with severe HFMD were significantly higher than those with mild symptoms [59]. In contrast, Gong et al. [60] showed upregulation of TLR2, TLR7, and TLR8 mRNA rather than TLR3, TLR4, TLR6, TLR9, or TLR10 at different time points in EV71-infected human primary monocyte-derived macrophages (MDMs). These results suggest that TLR signaling interacts with EV71 infection (see Figure 4 and Table 1).

## 9. EV71-Associated NOD-Like Receptor (NLR) Signaling 

NLRs, members of the pattern recognition receptor (PRR) family, are cytosolic counterparts of TLRs, which sense intracellular PAMPs and DAMPs [61]. Activation of some NLRs leads to the assembly of inflammasomes, which are large multiprotein complexes mediating the activation of inflammatory caspases. The best known inflammasome-forming NLRs are NLR family pyrin domain containing protein-1 (NLRP1), NLRP3, and NLR family caspase recruitment domain-containing protein-4 (NLRC4) [62]. Increasing evidence suggests that EV71 infection triggers inflammasome-mediated signaling [63,64,65]. EV71 3D^pro^ interacts with NLRP3 to facilitate the assembly of inflammasome complexes by forming a 3D^pro^-NLRP3-ASC ring-like structure, which results in the activation of interleukin (IL)-1β [63]. While another study indicated that EV71 counteracts inflammasome activation through the cleavage of NLRP3 by 2A^pro^ and 3C^pro^ [65]. Additionally, this study found that EV71 3C^pro^ interacted with NLRP3 and suppressed IL-1β secretion when expressed in mammalian cells [65]. IL-1β and IL-18 are regulated by inflammasome activation (e.g., NLRP3 inflammasome) [62]. A recent study found that IL-18 exhibited a protective role against EV71 infection in mice, and administration of recombinant IL-18 could reverse EV71 infection-induced pathogenesis in vivo [64]. Overall, EV71 infection induces NLRP3 activation-associated signaling pathways, which may further regulate inflammatory responses and antiviral innate immunity (see Figure 4).

## 10. EV71 Infection Activates Epidermal Growth Factor Receptor (EGFR) Signaling

EGFR belongs to a family of receptor tyrosine kinases which includes three other members (erbB2/HER-2, erbB3/HER-3, and erbB4/HER-4). EGFR is anchored in the cytoplasmic membrane and composed of an extracellular ligand-binding domain, a short hydrophobic transmembrane region, and an intracytoplasmic tyrosine kinase domain. EGFR becomes activated by receptor overexpression (mainly in cancer) as well as ligand-dependent and ligand-independent mechanisms in response to a variety of stimuli [66]. It has been widely appreciated that EGFR-dependent signaling plays an important role in the pathogenesis of inflammation through regulation of pro-inflammatory genes [67,68]. The cytoplasmic Src (c-Src) kinase is known to trans-activate EGFR. It has been shown that EV71 infection stimulates cyclooxygenase-2 (COX-2) expression and prostaglandin E2 (PGE_2_) release following transactivation of EGFR by c-Src in EV71-infected cells [69]. Another study indicated that EV71 infection induces ROS generation and NADPH oxidase activation through integrin β1/EGFR, in turn modulating EV71 replication in SK-N-SH cells [70]. Additionally, the attenuation of the IFN signal pathway resulting from EGFR phosphorylation by reducing the expression of miR-27a might be one of the main reasons for the promotion of EV71 replication by the activation of MEK/ERK [71]. Thus, EV71 infection induces inflammatory responses and EV71 replication through EGFR signaling (see Figure 2).

## 11. EV71 Activates Mitogen-Activated Protein Kinase (MAPK) Signaling

The MAPK signaling pathway is the most characterized of all the signal transduction pathways. It regulates various physiological processes in cells—including inflammation, stress, cell growth, cell development, differentiation, and death—through multiple substrates including phosphorylated transcription factors and enzymes [72]. MAPK signal transduction occurs via sequential phosphorylation of MAPKKK (mitogen-activated protein kinase kinase kinase), MAPKK (mitogen-activated protein kinase kinase) and MAPK. To date, six MAPK sub-families have been identified in mammalian cells: JNK1/2/3, ERK1/2, p38MAPK (p38 α/β/γ/δ), ERK7/8, ERK3/4 and ERK5/big MAP kinase 1 (BMK1) [73]. Many studies have indicated that MAPK pathways can be activated by EV71 infection [11,50,74,75,76]. Xie et al. reported that the p38, ERK1/2, and JNK1/2 pathways are activated during EV71 infection in human tonsillar epithelial cells, resulting in an over-expression of IL-8, IL-1β, IL-6 and IL-12p40 [49]. Similar results were also found in human intestinal epithelial cells, vascular smooth muscle cells, and immature DCs [52,75,77]. EV71 infection activates both JNK1/2 and p38, with subsequent phosphorylation of downstream transcription factors c-Fos and c-Jun in immature DCs. This leads to enhanced production of IL-2, IL-6, IL-10, and TNF-α. In addition, the administration of JNK1/2 and p38 inhibitors (e.g., SP600125 and SB203580) prevents EV71 replication and inflammatory cytokine production (e.g., IL-6, IL-10, and TNF-α) [75]. PD169316, another p38 inhibitor, reduces EV71-induced apoptosis in vitro, implying the activation of p38 by EV71 infection [11]. In vivo experiments showed that PD169316 can inhibit the replication of EV71, reduce tissue damage, and block the release of proinflammatory cytokines (e.g., MCP-1 and TNF-α) [11]. Similarly, another study reported that EV71 infection led to intrinsic apoptosis and induction of p38-mediated proinflammatory cytokines in human astrocytes [78]. Leong et al. found that the phosphorylation of p38 was stimulated by EV71 infection following misshapen/ Nck-interacting kinase (NIK)-related kinase (MINK) activation [76]. TAK1 is a member of MAPKKK. 3C^pro^ can influence TAK1 complex proteins to suppress EV71 infection-induced cytokine production. Therefore, on the one hand, EV71 infection triggers inflammatory response through activating MAPK pathways [79,80]. On the other hand, EV71 may inhibit MAPK pathways (TAK1 signaling) to interact with inflammatory response (see Figure 2).

## 12. EV71 Induces Phosphatidylinositol 3-Kinase (PI3K) Signaling

PI3K signaling is known to regulate cellular proliferation and growth [81], and play a critical role in triggering inflammatory reactions through the activation of the downstream protein kinase Akt [82,83]. It has been suggested that EV71 activates the PI3K/Akt pathway which further regulates proinflammatory cytokine transcription (e.g., IL-8, IL-1β, IL-6 and IL-12p40) [49]. Activation of PI3K/Akt may be associated with the secretion of inflammatory cytokines in human RD cells [84,85]. EV71-induced Akt phosphorylation is associated with a PI3K-dependent mechanism [86]. Wortmannin, a PI3K-specific inhibitor, was shown to inhibit EV71 infection-induced PI3K/Akt activation in vascular smooth muscle cells [52]. Additionally, EV71-induced COX-2 expression and PGE2 production is mediated by the activation of a c-Src/PDGFR/PI3K/Akt/p42/p44 MAPK pathway to initiate the expression of a transcription factor of AP-1 in rat brain astrocytes [51]. In summary, EV71 infection can activate the PI3K/Akt pathway, further regulating inflammatory response (see Figure 2).

## 13. EV71 Activates Calcium (Ca^2+^)-Dependent Signaling

Ca^2+^ is a ubiquitous second messenger which regulates various reactions in eukaryotic cells [87]. A previous study demonstrated that elevated levels of mitochondrial Ca^2+^ are detected in EV71-infected cells, and calpain activation via Ca^2+^ flux plays an essential role in eliciting an apoptosis-inducing factor (AIF)-mediated, caspase-independent, apoptotic pathway during EV71 infection. Administration of ruthenium red, a mitochondrial Ca^2+^ influx inhibitor, significantly blocks calpain activation and AIF cleavage in EV71-infected cells [88]. Calmodulin-dependent protein kinase II (CaMKII) is necessary for Ca^2+^ homeostasis. The EV71 VP1 protein can activate CaMKII. If activated, it then phosphorylates the N-terminal domain of vimentin on serine 82, and in turn plays a structural role for viral replication [89]. Taken together, EV71 infection facilitates EV71 replication and EV71-induced host cell apoptosis by activating Ca^2+^—dependent signaling (see Figure 2).

## 14. Conclusions and Future Perspectives

In summary, antiviral and inflammatory cellular signaling pathways play a critical role in EV71-induced pathogenesis. It is apparent that RLRs, TLRs, and NLRs sense dsRNA or endogenous DNA derived from EV71 and EV71-induced host cell death to initiate antiviral innate immunity and inflammatory response by inducing downstream IRF-dependent signaling and/or NF-κB signaling. EV71-encoded proteases are antagonists of IFN, RIG-I, MDA5, IRF, TLR, and NLR-dependent signaling pathways, which provides the molecular mechanisms for evasion of the innate immune response during EV71 infection. Future research is required to uncover more details surrounding the nucleation of RLR signaling at the interface between the mitochondria and mitochondria-associated membranes. These future studies will likely identify new mitochondrial molecules that regulate MAVS signaling. A more in-depth understanding of the mechanisms by which TLRs and NLRs function as both signaling molecules and antiviral effectors will promote a clearer characterization of the interplay between PRRs and the innate immune system. Given the limited clinical studies and in vivo studies, we cannot conclude that controlling 2A^pro^, 2C^pro^, 3C^pro^, and 3D^pro^ synthesis, and dsRNA accumulation are underlying antiviral strategies for EV71 infection-associated HFMD. Additionally, the evidence linking 3C^pro^ with RIG-I and MDA5 is not clear yet. Further structural and functional characterizations of EV71 proteins are needed to understand how these viral virulence factors interact with the human immune system, which will be useful in developing a vaccine. Further understanding of these interactions, which future research should prioritize, will lead to broad implications for clinicians and overall improvements in public health as we grapple with the burdens of HFMD-derived morbidity and mortality.

On the other hand, EV71 infection induces host cell ROS accumulation which activates specific cell surface receptors (e.g., EGFR), intracellular kinases (e.g., MAPK, PI3K), and transcription factors (e.g., NF-κB, AP-1) to induce proinflammatory cytokine production. Additionally, Ca^2+^-dependent signaling is required for EV71-induced host cell apoptosis and EV71 replication. Further research is required to uncover more details surrounding the nucleation of RLR signaling at the interface between the mitochondria and mitochondria-associated membranes, which will probably identify new mitochondrial molecules that regulate MAVS signaling. A more in-depth understanding of the mechanisms by which TLRs and NLRs function as both signaling molecules and antiviral effectors will promote a clearer characterization of the interplay between PRRs and the innate immune system. In addition, further investigation into the regulation of mitochondria-derived ROS during EV71 infection is warranted, as these molecules appear to influence several important aspects of inflammatory response. The cytokine storm has been confirmed in the process of EV71 infection-severe inflammatory syndrome (e.g., brainstem encephalitis, aseptic meningitis, encephalitis, acute flaccid paralysis, myocarditis, and even fatal pulmonary edema) [1]. Whether these local inflammatory responses come from EV71 infection-induced inflammatory cellular signaling pathways or immune cell response is not fully clarified. The cross effect of proinflammatory cytokines derived from immune cell response and inflammatory cellular signaling pathways may increase pulmonary vascular permeability, and even edema. The manner in which these findings will inform specific anti-inflammation strategies, thus far rather generically targeted, will be another field for ongoing investigation and innovation. Additionally, finding out whether Ca^2+^-dependent signaling affects skeletal muscle cell and cardiomyocyte function will be useful to reveal the novel mechanism underlying EV71 infection-induced muscle damage and cardiac disease.

## Figures and Tables

**Table 1:** EV71 infection-associated toll-like receptors (TLRs). Abbreviations employed: monocyte-derived macrophages (MDMs), peripheral blood mononuclear cells (PBMCs), DCs, plasmacytoid dendritic cells (pDCs).

TLRs	Ligand	Cell Type	*Species*	References
TLR?	No data	Intestinal epithelial cells	*Human*	[44]
TLR2	No data	MDMs	*Human*	[60]
TLR3	No data	DCs, PBMCs	*Mouse, Human*	[26,60]
TLR4	PAMPs (dsRNA?)	DCs, PBMCs	*Mammalian cells, Human*	[57,60]
TLR7	DAMPs?	16HBE, MDMs, PBMCs	*Human*	[41,58,60]
TLR8	No data	MDMs	*Human*	[60]
TLR9	Endogenous DNA	pDCs	*Mouse*	[13]
